# Promoter and Terminator Optimization for DNA Methylation Targeting in *Arabidopsis*

**DOI:** 10.3390/epigenomes4020009

**Published:** 2020-06-12

**Authors:** Jason Gardiner, Jenny M. Zhao, Kendall Chaffin, Steven E. Jacobsen

**Affiliations:** 1 Department of Molecular, Cell, and Developmental Biology, University of California Los Angeles, CA 90095, USA; jasonlgardiner@ucla.edu (J.G.); jennyzhao@ucla.edu (J.M.Z.); 2 Institute for Society and Genetics, University of California, Los Angeles, CA 90095, USA; kchaffin@g.ucla.edu; 3 Howard Hughes Medical Institute, University of California Los Angeles, Los Angeles, CA 90095, USA

**Keywords:** epigenetics, DNA methylation, targeting, zinc finger, *Arabidopsis*, plant, transcription, promoter, terminator, RNA-directed DNA methylation (RdDM), silencing

## Abstract

DNA methylation is an important epigenetic mark involved in gene regulation and silencing of transposable elements. The presence or absence of DNA methylation at specific sites can influence nearby gene expression and cause phenotypic changes that remain stable over generations. Recently, development of new technologies has enabled the targeted addition or removal of DNA methylation at specific sites of the genome. Of these new technologies, the targeting of the catalytic domain of *Nicotiana tabacum* DOMAINS REARRANGED METHYLTRANSFERASE 2 (ntDRM2cd) offers a promising tool for the addition of DNA methylation as it can directly methylate DNA. However, the methylation targeting efficiency of constructs using ntDRM2cd thus far has been relatively low. Previous studies have shown that the use of different promoters or terminators can greatly improve genome-editing efficiencies. In this study, we systematically survey a variety of promoter and terminator combinations to identify optimal combinations to use when targeting the addition of DNA methylation in *Arabidopsis thaliana*.

## 1. Introduction

**Figure 1:**
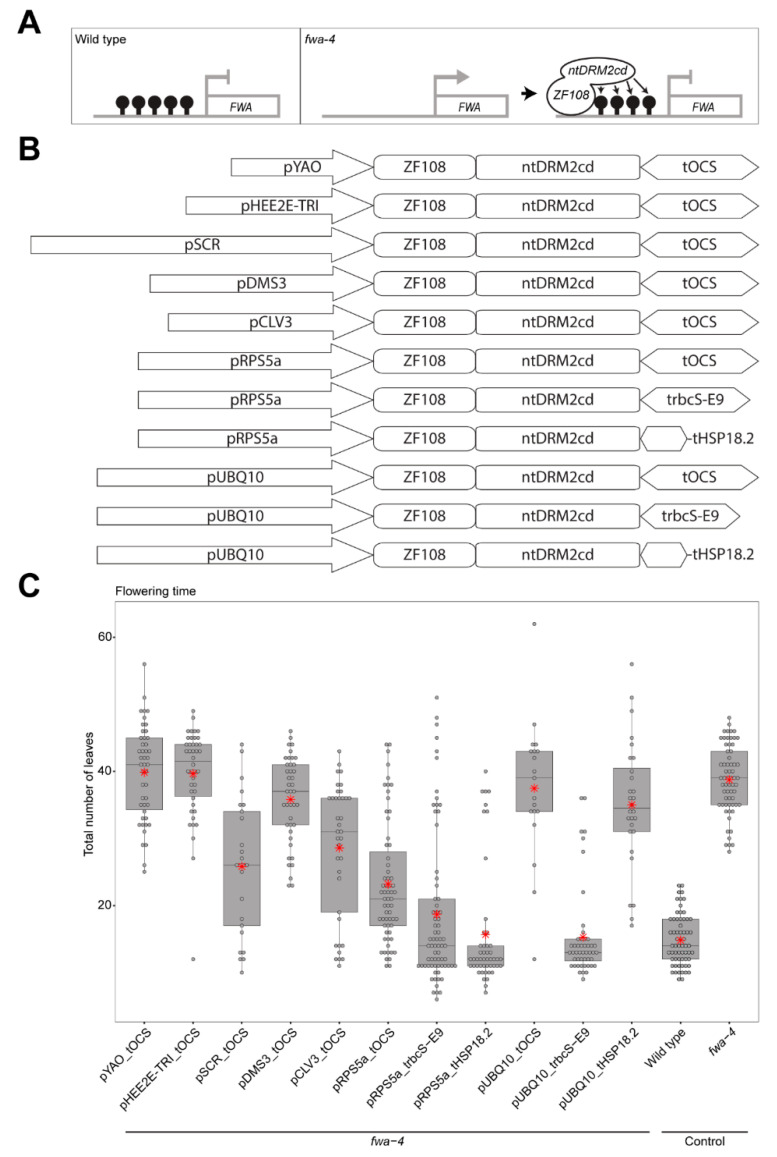
Use of different promoter and terminator combinations can change the efficiency of targeted DNA methylation. (**A**) Schematic representation of the *FLOWERING WAGENINGEN* (*FWA*) promoter region in wild type (where the promoter region is methylated and *FWA* gene expression is silenced), and the *FWA* promoter region in the *fwa-4* epiallele (where the promoter region is unmethylated and the *FWA* gene is expressed) being bound by the ZF108-ntDRM2cd fusion protein that adds methylation and silences *FWA* gene expression. (**B**) Schematic representation of all constructs used in this study. (**C**) Boxplot depicting the flowering time of the T1 population for each of the constructs in an *fwa-4* background, along with wild type, and *fwa-4* controls. Dots represent individual T1 plants. Red stars represent the mean leaf number for each population.

DNA methylation is an important epigenetic modification that can regulate gene expression and is typically associated with transcriptional silencing [1,2]. For example, in wild type *Arabidopsis* plants, DNA methylation in the promoter of the *FLOWERING WAGENINGEN* (*FWA*) gene silences *FWA* expression [3]. When this methylation is lost or removed from the *FWA* promoter region, as in the *fwa*-4 epiallele, there is an upregulation of *FWA* expression which increases the duration of the vegetative development phase, and causes plants to flower later than wild type [3,4]. This phenotype makes the *FWA* locus an ideal target to optimize new tools for the targeted manipulation of DNA methylation. Previously, the study of the function of DNA methylation required the genome-wide removal of DNA methylation through the mutation or chemical inhibition of components of the DNA methylation maintenance pathways [5,6,7,8,9]. With the advent of new targeting technologies, it is now possible to manipulate DNA methylation levels in a site-specific manner, in order to study the direct effects of methylation at high resolution [4,10,11,12]. An example of this is the fusion of a zinc finger designed to bind to the *FWA* promoter (ZF108) with components of the RNA directed DNA Methylation (RdDM) pathway, responsible for de novo methylation (such as: SU(VAR)3-9 HOMOLOG 2 (SUVH2), NUCLEAR RNA POLYMERASE D1A (NRPD1), RNA-DEPENDENT RNA POLYMERASE 2 (RDR2), SAWADEE HOMEODOMAIN HOMOLOG 1 (SHH1), DEFECTIVE IN MERISTEM SILENCING 3 (DMS3), RNA-DIRECTED DNA METHYLATION 1 (RDM1), SU(VAR)3-9 HOMOLOG 9 (SUVH9), and DOMAINS REARRANGED METHYLTRANSFERASE 2 (DRM2)) [10,11]. These fusions were able to target the addition of DNA methylation at the *FWA* promoter and rescue the late flowering phenotype of *fwa-4* [10,11]. Because the targeting of the catalytic domain of *Nicotiana tabacum* DRM2 methyltransferase (ntDRM2cd) represents the direct recruitment of a methyltransferase to DNA, it is a promising candidate for continued development of targeted DNA methylation tools, as it should be portable to other species (Figure 1A). However, while ZF108-ntDRM2cd was able to cause methylation and silencing of *FWA* in some individual plants, only approximately 10–20% of T1 plants displayed this phenotype [11]. Thus, the targeting of ntDRM2cd offers an excellent tool for the study of improvements that can increase the efficiency of DNA methylation targeting.

Other studies optimizing the targeting of genome-editing constructs have found changes in the promoter and terminator sequences can have a significant effect on the efficiency of targeted editing [13,14,15]. This suggests that the selection of transcriptional control components is an important consideration during construct design. In this study, we test the effect of different promoter and terminator combinations on the efficiency of ntDRM2cd targeted DNA methylation, and show that certain combinations dramatically increase the efficiency of methylation targeting. These findings will advance epigenetic research in plants and could be useful for future crop improvement efforts.

## 2. Results

Previous studies have used ZF108 to target effector proteins that restore DNA methylation at the *FWA* promoter in the *fwa-4* epiallele background with varying degrees of success [4,10,11]. To improve the efficiency of targeted genome-editing, previous studies have used promoters of genes that are active in meristematic tissue or the egg cell [14,15,16]. To test if these promoters can increase the DNA methylation targeting efficiency of ntDRM2cd, the *RIBOSOMAL PROTEIN 5A* promoter (pRPS5a), *SCARECROW* promoter (pSCR), *DMS3* promoter (pDMS3), *YAO* promoter (pYAO), *HEE2E-TRI* promoter (pHEE2E-TRI), or *CLAVATA 3* promoter (pCLV3) were cloned upstream of a ZF108-NLS-ntDRM2cd cassette also containing an *OCTOPINE SYNTHASE* terminator (ZF108-NLS-ntDRM2cd_tOCS), and transformed into an *fwa-4* background, and compared with the efficiency of the ubiquitously expressed *UBIQUITIN 10* promoter (pUBQ10) driving the same cassette [14,15,16,17,18,19,20,21,22,23] (Figure 1B,C). We counted the total number of rosette and cauline leaves produced during the vegetative phase of development for each T1 plant as a measure of flowering time. Of the constructs tested, pRPS5a_tOCS produced the highest proportion of early flowering plants with an average leaf count of 23.19 (S.E. = ±1.14); pSCR_tOCS and pCLV3_tOCS also produced a high proportion of early flowering plants with average leaf counts of 25.80 (S.E. = ±1.98) and 28.57 (S.E. = ±1.63), respectively; and pYAO_tOCS, pHEE2E-TRI_tOCS, and pDMS3_tOCS produced average leaf counts similar to the pUBQ10_tOCS (39.85, S.E. = ±1.03; 39.64, S.E. = ±1.05; 35.80, S.E. = ±1.00; and 37.47, S.E. = ±2.35, respectively) (Figure 1C). These results suggest that for constructs targeting DNA methylation, pRPS5a, pSCR and pCLV3 are more efficient promoters than pUBQ10.

Previous studies have also shown that the terminator sequence downstream of the coding sequence can also impact the efficiency of targeting constructs [14,15]. For instance, the *Pisum sativum RUBISCO SMALL SUBUNIT E9* terminator (trbcS-E9) increased the efficiency of gene editing compared to a *Nopaline Synthase* terminator (tNOS) when used in combination with *pHEE2E-TRI* [15]. Similarly, constructs using the *HEAT SHOCK PROTEIN 18.2* terminator (tHSP18.2) were found to be more efficient at targeting CAS9-based editing than constructs using a *35s* terminator (t35s) [14]. To test if these terminators can also improve the efficiency of targeted DNA methylation, the trbcS-E9 and tHSP18.2 terminators were used in place of the tOCS terminator in the top performing promoter construct, pRPS5a_tOCS (Figure 1B). We analyzed the flowering time as above for each T1 plant. Of the constructs tested, pRPS5a_trbcS-E9 and pRPS5a_tHSP18.2 combinations produced a lower average leaf count in T1 plants compared to the pRPS5a_tOCS construct (18.69, S.E. ± 1.35 and 15.67, S.E. ± 1.30, respectively) (Figure 1C), indicating that using trbcS-E9 and tHSP18.2 in combination with pRPS5a can further increase DNA methylation targeting efficiency.

While targeting DNA methylation using the *RPS5a* meristematic promoter was the most successful in terms of changes in flowering time, for some applications, such as analysis in non-meristematic tissue of T1 plants, it would be useful to have a more efficient ubiquitously expressed targeting construct. To achieve this, trbcS-E9 and tHSP18.2 terminators were also used to replace the tOCS terminator of pUBQ10_tOCS. We found that the pUBQ10_trbcS-E9 combination successfully produced a lower average leaf count in T1 plants (15.31, S.E. ± 0.97) as compared to the pUBQ10_tOCS combination (Figure 1C), while the pUBQ10_tHSP18.2 was similar to the original pUBQ10_tOCS combination (35, S.E. ± 1.77) (Figure 1C). This indicates that using trbcS-E9 in combination with pUBQ10 can increase the efficiency of targeted DNA methylation compared to tOCS.

**Figure 2:**
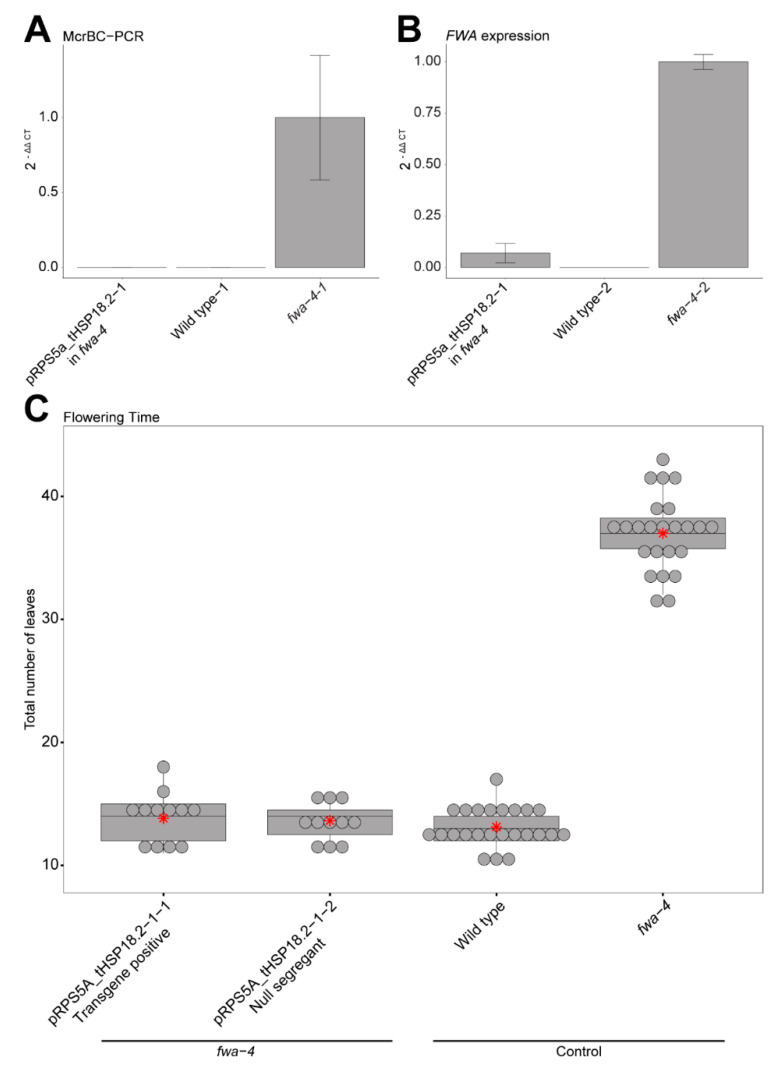
Confirming DNA methylation, *FWA* expression and heritability in the pRPS5a_tHSP18.2 background. (**A**) Bar graph showing relative methylation levels in an early flowering T1 plant containing the pRPS5a_tHSP18.2 construct in an *fwa-4* background, wild type, and *fwa-4* by McrBC-PCR. Mean values ± S.E. (*n* = 3, technical replicates). (**B**) Bar graph showing relative expression of *FWA* in a T1 plant containing the pRPS5a_tHSP18.2 construct in an *fwa-4* background, wild type, and *fwa-4* by RT-qPCR. Mean values ± S.E. (*n* = 3, technical replicates). (**C**) Boxplot depicting the flowering time of pRPS5a_tHSP18.2 T3 populations that have either maintained the transgene (transgene positive) or had the construct segregated away (null segregant) in an *fwa-4* background, along with wild type, and *fwa-4* controls. Dots represent individual T1 plants. Red stars represent the mean leaf number for each population.

Previous studies demonstrated that the flowering time change induced by the ZF108-ntDRM2cd fusion in the *fwa-4* background is associated with targeted DNA methylation to the *FWA* promoter and an associated decrease in *FWA* RNA expression [10,11,12]. To confirm that changes in flowering time seen for the best performing pRPS5a_tHSP18.2 construct were also associated with hypermethylation and gene silencing, we used McrBC-PCR to examine methylation and RT-qPCR to examine expression in an early flowering plant containing the pRPS5a_ tHSP18.2 transgene (Figure 2A,B). As expected, we found an increased amount of methylation at the *FWA* promoter (Figure 2A) and a decrease in *FWA* expression (Figure 2B).

Similarly, previous studies have shown that when a sufficient amount of methylation is targeted to the *FWA* promoter in an *fwa-4* background such that a decrease in *FWA* expression is observed and the late flowering phenotype of *fwa-4* is rescued, this change is largely heritable [10,11,12]. To test the heritability of the earlier flowering phenotype in the top pRPS5a combination, pRPS5a_tHSP18.2, we counted the total leaf number of T3 plants that had inherited the transgene in the T3 generation or T3 plants in which the transgene had segregated away (null segregants), as a measure of flowering time. Both T3 populations maintained a low average leaf count regardless of whether the construct was maintained or not (13.83, S.E. = ±0.58 for the transgene positive population and 13.64, S.E. = ±0.47 for the null segregant population) (Figure 2C). Thus, consistent with previous studies, the early flowering phenotype driven by the pRPS5a_tHSP18.2 construct causes methylation and silencing of *FWA* that is heritable independent of the continued presence of the transgene [10,11].

## 3. Discussion

The development of epigenetic targeting technologies utilizing zinc fingers, CRISPR or TAL effectors has created the opportunity to investigate the effects of epigenetic marks such as DNA methylation on specific loci in the genome [24]. Using these technologies and components of the RdDM pathway we have recently demonstrated the ability to target the addition of DNA methylation in *Arabidopsis thaliana* [10,11,12].

In this study, we explored the effect of different transcriptional components on the efficiency of targeted DNA methylation. We found that using meristem-specific promoters greatly increases the efficiency of targeting of DNA methylation and that utilizing different terminator sequences in the most efficient promoter construct (pRPS5a_tOCS) could increase the efficiency further (Figure 1C). Furthermore, utilizing different terminator sequences greatly increased the efficiency of the ubiquitously expressed promoter variant, pUBQ10_tOCS, although not as consistently as with the pRPS5a constructs (Figure 1C). While all combinations using pRPS5a worked better than the original pUBQ10_tOCS construct, suggesting that pRPS5a robustly drives the expression of ZF108-NLS-ntDRM2cd regardless of terminator selection, pUBQ10 showed an improved efficiency only when combined with trbcS-E9 terminator, suggesting that terminator selection is an important consideration when using the pUBQ10 promoter to drive expression of synthetic constructs (Figure 1C).

This study adds to the growing body of knowledge used to guide the creation of efficient synthetic tools for the targeted manipulation of the epigenetic landscape. The utilization of the catalytic domain of *Nicotiana tabacum* DRM2 is especially attractive because it should be portable to other crop species. For instance, this domain was cloned from the *Nicotiana tabacum DRM2* gene, but works efficiently in *Arabidopsis*. Because the domain contains catalytic activity, this approach is likely to also work in other more distantly related plants such as monocot species, and is also likely to work in animals or fungi.

## 4. Materials and Methods

### 4.1. ZF Design and Cloning

pUBQ10_ZF108_tOCS was previously created by Gallego-Bartolomé et al. [11]. A NLS-ntDMR2cd coding sequence was then delivered into the pUBQ10_ZF108_tOCS by an LR reaction (Invitrogen, Carlsbad, CA, USA) resulting in pUBQ10_ZF108-NLS-ntDRM2cd_tOCS (pUBQ10_tOCS). To create vectors with different promoters, the existing pUBQ10 promoter was digested out of the pUBQ10_ZF108-NLS-ntDRM2cd_tOCS vector using MluI (New England Biolabs, Inc., Ipswich, MA, USA). Promoters were then amplified with their respective primers (Tables S1 and S2) from genomic DNA or from pre-existing plasmid DNA (pHEE2E-TRI-Addgene: 71288) and cloned into the MluI restriction site using In-Fusion (Takara Bio USA, Inc., Mountain View, CA, USA). To create vectors with different terminators the existing OCTOPINE SYNTHASE terminator (tOCS) was digested out of the pRPS5a_ ZF108-NLS-ntDRM2cd_tOCS vector using BstXI (New England Biolabs, Inc.). Terminators were then amplified with their respective primers (Tables S2 and S3) from genomic DNA or from pre-existing plasmid DNA (pHEE2E-TRI-Addgene: 71288) and cloned into the BstXI restriction site using In-Fusion (Takara Bio USA, Inc.). pHEE2E-TRI was a gift from Qi-Jun Chen (Addgene plasmid # 71288; http://n2t.net/addgene:71288; RRID:Addgene_71288). The resulting pRPS5a_ZF108-NLS-ntDRM2cd_trbcS-E9 (pRPS5a_trbcS-E9) and pRPS5a_ZF108-NLS-ntDRM2cd_tHSP18.2 (pRPS5a_tHSP18.2) were then digested with MluI (New England Biolabs, Inc.) to remove the pRPS5a promoter and the pUBQ10 promoter was amplified using pUBQ10-specific primers (Tables S1 and S2) from *Arabidopsis* genomic DNA and cloned into the MluI restriction site using In-Fusion (Takara Bio USA, Inc.) to create pUBQ10_ZF108-NLS-ntDRM2cd_trbcS-E9 (pUBQ10_trbcS-E9) and pUBQ10_ ZF108-NLS-ntDRM2cd_tHSP18.2 (pUBQ10_tHSP18.2).

### 4.2. Plant Material, Growth Conditions and Flowering Time Assay

All plants were grown under long day conditions. The *Arabidopsis* Columbia-0 ecotype (Col-0) was used exclusively in this study. The origin of the *fwa-4* epiallele was previously described [10]. T1 transgenic plants were obtained via *Agrobacterium*-mediated floral dipping and selected using 0.5× MS medium (MP Biomedicals, LLC, Irvine, CA, USA) + 10 μg/mL glufosinate ammonium (PlantMedia, Dublin, OH, USA) + 0.1% of Plant Preservative Mixture (Plant Cell Technology, N.W. Washington DC, USA) + Cefotaxime 100 μg/mL (Cayman Chemical, Ann Arbor, Michigan, USA). In T2 and T3 generations, transgenic plants were selected using 0.5× MS medium (MP Biomedicals, LLC) + 10 μg/mL glufosinate ammonium (PlantMedia). Flowering time was scored by counting the total number of rosette and cauline leaves after bolting.

### 4.3. McrBC-PCR

CTAB-extracted genomic DNA was digested using the McrBC restriction enzyme (New England Biolabs, Inc.) for 4 h at 37 °C. As a control, an equal amount of DNA was incubated in digestion buffer without McrBC enzyme for 4 h at 37 °C. Quantitative PCR (qPCR) of the resulting digested and undigested samples was performed using 5′-ttgggtttagtgtttacttg-3′ and 5′-gaatgttgaatgggataaggta-3′ oligos specific for the *FWA* promoter. The *C*_t_ values collected from digested samples were first normalized to *C*_t_ values collected from nondigested samples and then to the *fwa-4* control background using the ∆∆*C*_t_ method.

### 4.4. Reverse Transcriptase-qPCR

RNA was extracted using Direct-zol RNA Miniprep kit (Zymo, Irvine, CA, USA). cDNA was prepared using the SuperScript III First-Strand Synthesis SuperMix (Invitrogen). qPCR of the resulting cDNA was conducted using oligos specific for *FWA*, 5′-ttagatccaaaggagtatcaaag-3′ and 5′-ctttggtaccagcggaga-3′, and oligos specific for the *ISOPENTENYL PYROPHOSPHATE:DIMETHYLALLYL PYROPHOSPHATE ISOMERASE 2* (*IPP2*) housekeeping gene, 5′-gtatgagttgcttctccagcaaag-3′ and 5′-gaggatggctgcaacaagtgt-3′. The *C*_t_ values collected from qPCR reactions using *FWA*-specific oligos are first normalized to *C*_t_ values collected from qPCR reactions using *IPP2*-specific oligos and then to the *fwa-4* control background using the ∆∆*C*_t_ method.

## Supplementary Materials

The following are available online at https://www.mdpi.com/2075-4655/4/2/9/s1, Table S1: Promoter sequences, Table S2: Primer sequences, Table S3: Terminator sequences.

## References

[1] Law J.A., Jacobsen S.E. (2010). Establishing, maintaining and modifying DNA methylation patterns in plants and animals. Nat. Rev. Genet..

[2] Zhang H., Lang Z., Zhu J.K. (2018). Dynamics and function of DNA methylation in plants. Nat. Rev. Mol. Cell Biol..

[3] Soppe W.J., Jacobsen S.E., Alonso-Blanco C., Jackson J.P., Kakutani T., Koornneef M., Peeters A.J.M. (2000). The late flowering phenotype of fwa mutants is caused by gain-of-function epigenetic alleles of a homeodomain gene. Mol. Cell.

[4] Gallego-Bartolomé J., Gardiner J., Liu W., Papikian A., Ghoshal B., Kuo H.Y., Zhao J.M.-C., Segal D.J., Jacobsen S.E. (2018). Targeted DNA demethylation of the *Arabidopsis* genome using the human TET1 catalytic domain. Proc. Natl. Acad. Sci. USA.

[5] Kankel M.W., Ramsey D.E., Stokes T.L., Flowers S.K., Haag J.R., Jeddeloh J.A., Riddle N.C., Verbsky M.L., Richards E.J. (2003). *Arabidopsis* MET1 cytosine methyltransferase mutants. Genetics.

[6] Baubec T., Pecinka A., Rozhon W., Mittelsten Scheid O. (2009). Effective, homogeneous and transient interference with cytosine methylation in plant genomic DNA by zebularine. Plant J..

[7] Taylor S.M., Jones P.A. (1982). Changes in phenotypic expression in embryonic and adult cells treated with 5-azacytidine. J. Cell. Physiol..

[8] Griffin P.T., Niederhuth C.E., Schmitz R.J. (2016). A Comparative Analysis of 5-Azacytidine- and Zebularine-Induced DNA Demethylation. G3.

[9] Ji L., Jordan W.T., Shi X., Hu L., He C., Schmitz R.J. (2018). TET-mediated epimutagenesis of the *Arabidopsis thaliana* methylome. Nat. Commun..

[10] Johnson L.M., Du J., Hale C.J., Bischof S., Feng S., Chodavarapu R.K., Zhong X., Marson G., Pellegrini M., Segal D.J. (2014). SRA- and SET-domain-containing proteins link RNA polymerase V occupancy to DNA methylation. Nature.

[11] Gallego-Bartolomé J., Liu W., Kuo P.H., Feng S., Ghoshal B., Gardiner J., Zhao J.M.C., Park S.Y., Chory J., Jacobsen S.E. (2019). Co-targeting RNA Polymerases IV and V Promotes Efficient De Novo DNA Methylation in *Arabidopsis*. Cell.

[12] Papikian A., Liu W., Gallego-Bartolomé J., Jacobsen S.E. (2019). Site-specific manipulation of *Arabidopsis* loci using CRISPR-Cas9 SunTag systems. Nat. Commun..

[13] Feng Z., Zhang Z., Hua K., Gao X., Mao Y., Botella J.R., Zhu J.K. (2018). A highly efficient cell division-specific CRISPR/Cas9 system generates homozygous mutants for multiple genes in *Arabidopsis*. Int. J. Mol. Sci..

[14] Tsutsui H., Higashiyama T. (2017). PKAMA-ITACHI vectors for highly efficient CRISPR/Cas9-mediated gene knockout in *Arabidopsis thaliana*. Plant Cell Physiol..

[15] Wang Z.-P., Xing H.-L., Dong L., Zhang H.-Y., Han C.-Y., Wang X.-C., Chen Q.-J. (2015). Egg cell-specific promoter-controlled CRISPR/Cas9 efficiently generates homozygous mutants for multiple target genes in *Arabidopsis* in a single generation. Genome Biol..

[16] Yan L., Wei S., Wu Y., Hu R., Li H., Yang W., Xie Q. (2015). High-Efficiency genome Editing in *Arabidopsis* Using YAO Promoter-Driven CRISPR/Cas9 System. Mol. Plant.

[17] Yadav R.K., Tavakkoli M., Xie M., Girke T., Reddy G.V. (2014). A high-resolution gene expression map of the *Arabidopsis* shoot meristem stem cell niche. Development.

[18] Kim J.Y., Yuan Z., Cilia M., Khalfan-Jagani Z., Jackson D. (2002). Intercellular trafficking of a KNOTTED1 green fluorescent protein fusion in the leaf and shoot meristem of *Arabidopsis*. Proc. Natl. Acad. Sci. USA.

[19] Weijers D., Franke-van Dijk M., Vencken R.J., Quint A., Hooykaas P., Offringa R. (2001). An *Arabidopsis* Minute-like phenotype caused by a semi-dominant mutation in a RIBOSOMAL PROTEIN S5 gene. Development.

[20] Yadav R.K., Girke T., Pasala S., Xie M., Reddy G.V. (2009). Gene expression map of the *Arabidopsis* shoot apical meristem stem cell niche. Proc. Natl. Acad. Sci. USA.

[21] Klepikova A.V., Kasianov A.S., Gerasimov E.S., Logacheva M.D., Penin A.A. (2016). A high resolution map of the *Arabidopsis thaliana* developmental transcriptome based on RNA-seq profiling. Plant J..

[22] Schindele P., Puchta H. (2020). Engineering CRISPR/Lb Cas12a for highly efficient, temperature-tolerant plant gene editing. Plant Biotechnol. J..

[23] Waese J., Fan J., Pasha A., Yu H., Fucile G., Shi R., Cumming M., Kelley L.A., Sternberg M.J., Krishnakumar V. (2017). ePlant: Visualizing and exploring multiple levels of data for hypothesis generation in plant biology. Plant Cell.

[24] Gallego-Bartolomé J. (2020). DNA methylation in plants: Mechanisms and tools for targeted manipulation. New Phytol..

