# Supplementary material for: Promoter and Terminator Optimization for DNA Methylation Targeting in Arabidopsis
Source: Epigenomes. 2020 Jun 12;4(2):9. doi: 10.3390/epigenomes4020009 (PMC8594676; doi:10.3390/epigenomes4020009)
Supplement: Supplementary file 1 [file epigenomes-04-00009-s001.pdf]

Table S1. Promoter sequences

Promoter	Sequence (5'-3')
pYAO (AT4G05410)	gatgggaaattcattgaaaaccctaaacccaaatcaacagctgcaattcaaaaggggactaattgacaaacaaaaattgataacaaata<br>gaggtagggggagagtttcgtacgcgacaatgagattgagctcttgaggacttgtgaagttgccaacgcacgagtgagtgacactggt<br>cggtttgtgagccgtaacaacgtagttccatgagctcatcttcctcttctttgtctccagggaatttgagttcgactttctacgcgagggccct<br>cgaggaagcttctagatttctgaatcgagctttcggaattttaacatagagaagttagagagagaatgaaaagccaaaggaggcgaaa<br>atcgaacaaggaagaagaaagacaactttcgacaaagactggtcggtcggttttggtagacaattgaaattagatggatggtccggttc<br>ggtatactataagattaaaaacagttttaaattcagctaaaccgaactcatttgattttattaaaccggaatcatccgattcgagtttgtaaaa<br>aataccgaaattgaaaacactaaacaaaaactgtattaaactgttactgaaataagagaatctcccaattcggtttacgtactactcttcag<br>aaatcagaaccaaaaattcagaaatcggattgaaccaaacttaaattgacggtccggttagtcttcggctctacaaattaaaggcccaagt<br>ttctgctttaaaagaacgaaatagttaatgggctcaaaccatagaccaggtaagtcatgggcttggttagtccgggtcaacccggtagac<br>ccgattcctgaagaaaacctagtggaaggtttaaagttgtaaactttccgaccaaataaacaaaatcgttttccagcttcttccgtcgccact<br>aaaccctgaggctaaacctagacgagtcaaagtgtaaaatcgttaaaccctaagagggagtgagagagagaagaatgaagtacaaca<br>acgagaagaagaaaggagtcgag
pHEE2E-TRI (Addgene:71288)	gaataaaagcatttgcgtttggtttatcattgcgtttatacaaggacagagatccactgagctggaatagcttaaaaccattatcagaacaa<br>aataaaccatttttgttaagaatcagagcatagtaaacaacagaaacaacctaagagaggtaacttgtccaagaagatagctaattatat<br>ctattttataaaagttatcatagtttgtaagtcacaaaagatgcaaataacagagaaactaggagacttgagaatatacattcttgtatatttg<br>tattcgagattgtgaaaatttgaccataagtttaaattcttaaaaagatatatctgatctaggtgatggttatagactgtaattttaccacatgtt<br>taatgatggatagtgacacacatgacacatcgacaacactatagcatcttatttagattacaacatgaaatttttctgtaatacatgtctttgta<br>cataatttaaaagtaattcctaagaaatatatttatacaaggagtttaaagaaaacatagcataaagttcaatgagtagtaaaaaccatata<br>cagtatatagcataaagttcaatgagtttattacaaaagcattggttcactttctgtaacacgacgttaaaccttcgtctccaataggagcgct<br>actgattcaacatgccaatatatactaaatacgtttctacagtcaaatgctttaacgtttcatgattaagtgactatttaccgtcaatcctttccca<br>ttcctcccactaatccaacttttaattactcttaaatcaccactaagctagtaacgcctatcatgaattagctctactaaatctagcaacctttca<br>aatttgcagtattgcaggtgtctctgtgtctttaaaatagttgccttatgatttcttcggtttcaagatgatcaaatagttatagatttcatgctca<br>cacatgctcattagatgtgtacatactttacttacccaaatctattttctcgcaaagattttgatggtaaagctgatttggttctattgaactaaat<br>caaacgagtttcagactgagtgattctaatccggcccattagcccctaaacagacccactaattacgcagcttttaatagagtaattacacct<br>agtttacccactaaaccactaagcactaattatctcacaatctaatgagcttccctcgtaattacttgggctttcactctaccatttatttgtaaca<br>gtcaagtctctactgtctctatataaactctctaaagttaacacacaattctcatcacaaacaaatcaaccaaagcaacttctactctttcttcttt<br>cgaccttatcaatctgttgagaa
pSCR (AT3G54220)	aattttgaatccattctcaaagctttgccaatctgcgttcgaaattcgaaaaccctaacgactttggagattcaaattgagccaaatctctatg<br>aaaagtggaaatttacctggaagtccgattgagaggagaggattgaccggagagagaccggagaaagatgggagtgaaggagaag<br>agattgggagacgaagagtaaccggacggcgagaagacgaggaaggtaatcggagtgatgcggtggagggaagagctgatttga<br>gagaagacatactccagactctgctcacggaatctgtaggagtcgccattgccatggacattggaatcgccattagattgtgatcctctgc<br>aacaaagcggattttgctggtgttgaatggataagggatagaggaagaggactttgtttatcagaaaccttttgatgggccttaatgggc<br>ctataaactgtaactctgtagcgctttgccaacaagagactttttaaggtttttgttgccaaacagatatttgcatttgggctatgtaatgttag<br>aattattttataatgtatgctattgctagatattgtttaagtgcatttgtgatttacaaacatttcattttattttggttttaatgagcatttctattat<br>agagactttgatgttaataaatggtgttctaagatatattaaaatattttatatactttcttaaaattggataaattttgggaaaatccttaatatc<br>agttaaattgaagataaagagtattaaaaaaaactatgtagtaaaatacatttcacatttttgtgtataatagtacatggtattcgttaagat<br>cactcaaaaattaacaaattaagtctaaaagggcagaaaagactattcaaatatggacttggagaaagacattcagctttttacgctgaga<br>aactttcatattgagccgtgtgtttgtgttgtgaagagaagtaataaaaaataatttgaagtgaaaaaggagaagaaaaaataagatcgt<br>agaaagcgtggatggtttcttcttgggttcactgccatgcgattattaaattggccatggggctagtgtttgacgtacaaaagtctaaaaatt<br>gtcagtcaaacaggtccaaaactttgtaagaaaaataatataataatagcaaattttctaaaaattgttaaaaaaagaacaaaagggaaa<br>agatgaggatgcagatgaaagcaaaatgtcaaacactagtttcagattttatcgggaactggggtttgacagttggtgtatgtatgtaatg<br>gcctctcatcaaaacatgtgcatctttttcctttttgttatttactgttttagctctacgtcttgtccaattcctctcaagtaaaatgcctttaatatg

	atactaatatacaaggggactaatgctttttcccttttcttatccttgttttgtctaaatctttacttggattcctttatttttctcctctctttagattagtacggtttaaggaataccatctttctaattttagcacaaaattgcaagttggtgccccatcttagtaagcacatcgtaccacactttgattgtgtgagagacttcttcatcccatctctcataccaaacctaaatcaaatgactagtggtgcaacctgctgactccatatgaccataactaataaatcggtttatgaatccaactcatgtagctctatagaatagaaacccattcatttcacataatgaactgaatctgacattttatttacatcatttactactcaattttgtaattagcaagatcatctttttcattattcaacaattttgatattccataatttattaactttgtcatacatcataatattctgaattttgttatatattgtaccggttccacgaaatagagctctattattatagaccaaacaaacaaaatattatcttcttgtggttagttcgagagagaggtcaagaagaaacgaaatggatcggcaaacggaagacgtcaaacacacaacgacgaacattttccgatcacccacctaatctcttcccatttttattatttttcaaaactcaaattaattaagaagaaaaaaacagaaacagagagagaaagagttaagatgaatagagatagaaagagtcattaaatgtacgaagcgacattcacaataattcgaaaggtggaagacgacttagatacggccaggcttcactgtcctcctcgtcctcctcaattacccctaacccctttttccgggattcatctccaacccacatccttccaaattctcaccccctcactgagtttttgcttttctcctcatcggagatcgtgaagacgatcaagtaatttaagaatcccaccattgataaaagagtctagcttttctactaccaaacctttttctgtttggaaattttcgattttggatttaacccttttcttaccttatttataaccatgcaatctcacgaccaacaacccttcaatctcc
pDMS3 (AT3G49250)	accgataaaatgaaaatcattaacggagtcacgtggggttcggttaaacgccgttaagtgtttttacgaagttcacgaggtttctacttttgtcttctcttattttaacgccaatcgcctttatttccatttgttataaagtcttctgacatttgtaatgatcgaattctgtatttacatatttgttatatcctatataaaatattatttgactttttcttaaattttgtggacttgtggacctattatgtttacatatttttgatgagaagatgccatatttcgaatatatatgtactagtgtaatctgaggaaattcaagttgaagccaatacatactttggaatttatatatttaaaaacaaaaaaaaactatattctataaataaatttacttatttactaattagtttgataaaattcagtttttaataatacttttatttgtatggttaaatatgctacttaacaaaatatttttaaatatatatattcatgagatttatccttcctttatatcgagcttaaaataaactattggtcacatgcatataatatgcatttatacataaagttctgatttatatgattaacattggtaacatgagtttagaagcaatatttatccgacacattttaaagaatgaacgaaaaatttaaaggtttcattcgtttaggtatacatacagaatttactcaagaactacaaaactttgatattaaaatatcaaatgatgccgatcatttgtcttaagatgtaaattaaaagagagaaaaaaaaacacaaaaatgaaaatgtgcagttgttttaataacggcgacctaactttctttatgaggacccaagacctacaccacctccaaaattggaccacctttcgatttcactactcattgagagtctcatacatccacacgagtagctaaactatttttttttaagcataaacaaagctggaattcatttttttttctttctaatgtaatcaaagcttaaattcaacattccttataaaatatgttataaatgataatgtttaggcaaaaacataacatcatgtcacatgatttttttttaatagaaaaccagtacggcgttcgacaatatcgatacacagagtcgttttcctaacgaacctaacgcatgacacagaactagttgaatttgtaagataaaaactggtttgacatgtatcggtatcgaactcaaatacgagaaattacctctggttcgggtttttcattatcaaactaatatgctacaaagtgagaaagtcaggctcagaaggaggtatgaagcacaaagcccacttggaatctccgaagattattgggccttaaataaggccagaaaatataattgaatgataaaaggaaacttttcttacatttttggcaaaatacgttccttttattattggtgtattttaatttagtttaattacttcacaacaattggctgtttttctccagaagaacactaaagacaaaaattaaaccctcacactcacagagaccggagaagaagcagagagaaagagagagagagagattcaccggtgataacattgaagcagaaagattctccggtgataacattgaagctgaggagca
pCLV3 (AT2G27250)	cggattatccataataaaaacaaaactagatacattttctaagtacattaacacataagaatatcatttttgtactaccaaaaaaaaagtaaaaacttatgtccatggtattattataattggtggtaaatcagtaaattagttgttagcaaaaaaataaaatttagttaaaaagtagtggcaccttattggcccaaaagagtaacgaagcaaaacggaatctgaaaactgttaaacttaaaagaatagataattgagatataatatcgtatgatcggacggctgtgatctgatgccatcggatgggcacgtgtcagaagtgtactccaggtatcattctctctgcctcatgacatcagccgatggtaccctcgatacggcgccggcaaggctcatataatccattcaatttatgtttttctgctagcaaatagatttgccattgatttgatactctcaataatattatccgattaaggtatcatccatattgttcaatataatttaagcatataactgtttccagattaaacaatataatttataagagcaactgtaatactttactttaaaagttttcaaatcagaatctcttttctttttctacaaatctggaaacaatttaatccatattgtttttgtttttttaccttctcatatttagatgctatttattattgtgacacaagcttagtttaatttcttgttggttacattttttttctaaatacacaaatattatatgtttaatattatttcacacatttcttagaagaaatctaaatacatgaaattatatttattaaaaagaagtctaaactatatatgtatttaaatggaattttaaatatgggtagtaatatagaaacaccattgatatattagagtatgtgccggtgccgtatacatactgttgtattatagtaacctatatatggtatacctagtcaatgtgtacggtatttttcatatgtattagttgtgaacctccacagcatgttagacttaggaattaattataactgaaccagaactttgagtctaattataccccctgccgttttatacaactttgaatattgattccagtggctaatttaaataagttaaaggaaacaaaaactgcaagtaagtgaaaaatacacaattgtaagtaaatgttagatatttaatttatttatagctaaatcatgaacaagttcgtataagatctagatatatgtaccatatacctttctatgcccactatatatacttactacacataatatataaaccaaccaaaatatttgaatagttaataactatgatacacgtttaggacaa

	ataaaattaaaaaataggagatcccatttctcgcccttgtaggcttacgctataaattggactgtccccttctcatttcattaccaaagtaaag<br>aacagtttctatatttctctctttatctctctcactcagtcactttctctctaaaa
pRPS5A(AT3G11940)	ccataatcgtgagtagatatattactcaacttttgattcgctatttgcagtgcacctgtggcgttcatcacatcttttgtgacactgtttgcactg<br>gtcattgctattacaaaggaccttcctgatgttgaaggagatcgaaagtaagtaactgcacgcataaccatttctttccgctctttggctcaa<br>tccatttgacagtcaaagacaatgtttaaccagctccgtttgatatattgtctttatgtgtttgttcaagcatgtttagttaatcatgcctttgatt<br>gatcttgaataggttccaaatatcaaccctggcaacaaaacttggagtgagaaacattgcattcctcggttctggacttctgctagtaaatta<br>tgtttcagccatatcactagctttctacatgcctcaggtgaattcatctatttccgtcttaactatttcggttaattaaagcacgaacaccattact<br>gcatgtagaagcttgataaactatcgccaccaatttatttttgttgcgatattgttactttcctcagtatgcagctttgaaaagaccaaccctctt<br>atcctttaacaatgaacaggtttttagaggtagcttgatgattcctgcacatgtgatcttggcttcaggcttaattttccaggtaaagcattatg<br>agatactcttatatctcttacatacttttgagataatgcacaagaacttcataactatatgctttagtttctgcatttgacactgccaaattcatta<br>atctctaatatctttgttgttgatctttggtagacatgggtactagaaaaagcaaactacaccaaggtaaaatactttgtacaaacataaact<br>cgttatcacggaacatcaatggagtgtatatctaacggagtgtagaaacatttgattattgcaggaagctatctcaggatattatcggtttat<br>atggaatctcttctacgcagagtatctgttattccccttcctctagctttcaatttcatggtgaggatatgcagttttctttgtatatcattcttcttc<br>ttctttgtagcttggagtcaaaatcggttccttcatgtacatacatcaaggatatgtccttctgaatttttatatcttgcaataaaaatgcttgtac<br>caattgaaacaccagctttttgagttctatgatcactgacttggttctaaccaaaaaaaaaaaaaatgtttaatttacatatctaaaagtaggttt<br>agggaaacctaaacagtaaaatatttgtatattattcgaatttcactcatcataaaaacttaaattgcaccataaaattttgttttactattaatg<br>atgtaatttgtgtaacttaagataaaaataatattccgtaagttaaccggctaaaaccacgtataaaccagggaacctgttaaaccggttctt<br>tactggataaagaaatgaaagcccatgtagacagctccattagagcccaaaccctaaatttctcatctatataaaaggagtgacattaggg<br>tttttgttcgtcctcttaaagcttctcgttttctctgccgtctctctcattcgcgcgacgcaaacgatcttcaggtgatcttctttctccaaatcctct<br>ctcataactctgatttcgtacttgtgtatttgagctcacgctctgtttctctcaccacagccg
pUBQ10(AT4G05320)	agtctagctcaacagagcttttaacccaaattggtacaatagaatacaactttagatcataattctcaaaagaaagagattccttagctattct<br>atctgccactccatttccttctcggcttgtatgcacaagcataaaatcctcaaacttgctaagtagatactttatgtcttggataattggattga<br>gacttgacaagcataactttcatgtaaccaaagacacaagttgctgagaatccacctcaaaaatgatcttcctataattgaatcgggataat<br>gacagcacagcccatctaagagcctccacttctacttccagcacgcttcttacttttaccacagctcttgcacctaaccataacaccttccctgt<br>atgatcgcgaagcacccaccctaagccacattttaatccttctgttggccatgccccatcaaagttgcacttaacccaagattgtggtggag<br>cttcccatgtttctcgtctgtcccgacggtgttgtggttggtgctttccttacattctgagcctctttccttctaatccactcatctgcatcttcttgt<br>gtccttactaatacctcattggttccaaattccctccctttaagcaccagctcgtttctgttcttccacagcctcccaagtatccaagggactaaa<br>gcctccacattcttcagatcaggatattcttgtttaagatgttgaactctatggaggtttgtatgaactgatgatctaggaccggataagttcc<br>cttcttcatagcgaacttattcaaagaatgttttgtgtatcattcttgttacattgttattaatgaaaaaatattattggtcattggactgaacacg<br>agtgttaaatatggaccaggccccaaataagatccattgatatatgaattaaataacaagaataaatcgagtcaccaaaccacttgccttttt<br>taacgagacttgttcaccaacttgatacaaaagtcattatcctatgcaaatcaataatcatacaaaaatatccaataacactaaaaaattaaa<br>agaaatggataatttcacaatatgttatacgataaagaagttacttttccaagaaattcactgattttataagcccacttgcattagataaatg<br>gcaaaaaaaaacaaaaaggaaaagaaataaagcacgaagaattctagaaaatacgaaatacgcttcaatgcagtgggacccacggtt<br>caattattgccaattttcagctccaccgtatatttaaaaaataaaacgataatgctaaaaaaatataaatcgtaacgatcgttaaatctcaacg<br>gctggatcttatgacgaccgttagaaattgtggttgtcgacgagtcagtaataaacggcgtcaaagtggttgcagccggcacacacgagt<br>cgtgtttatcaactcaaagcacaaatacttttcctcaacctaaaaataaggcaattagccaaaaacaactttgcgtgtaaacaacgctcaata<br>cacgtgtcattttattattagctattgcttcaccgccttagctttctcgtgacctagtcgtcctcgtcttttcttcttcttcttctataaaacaataccc<br>aaagagctcttcttcttcacaattcagatttcaatttctcaaaatcttaaaaactttctctcaattctctctaccgtgatcaaggtaaatttctgtgt<br>tccttattctctcaaaatcttcgattttgttttcgttcgatcccaatttcgtatatgttctttggtttagattctgttaatcttagatcgaagacgattt<br>tctgggtttgatcgttagatatcatcttaattctcgattagggtttcatagatatcatccgatttgttcaaataatttgagttttgtcgaataatta<br>ctcttcgatttgtgatttctatctagatctggtgttagtttctagtttgtgcgatcgaatttgtcgattaatctgagtttttctgattaacag

Table S2. Primer sequences

Primer	Sequence (5'-3')
pYAO FWD	gaggcgcgccacgcgtgatgggaaattcattgaaaaccc
pYAO REV	ccaaccatggacgcgtctcgactcctttcttcttctcg
pHEE2E-TRI FWD	gaggcgcgccacgcgtgaataaaagcatttgcgtttg
pHEE2E-TRI REV	ccaaccatggacgcgtttctcaacagattgataaggtc
pSCR FWD	gaggcgcgccacgcgtaattttgaatccattctcaaa
pSCR REV	ccaaccatggacgcgtggagattgaagggttgttgg
pDMS3 FWD	gaggcgcgccacgcgtaccgataaaatgaaaatcattaac
pDMS3 REV	ccaaccatggacgcgttgctcctcagcttcaatgttatc
pCLV3 FWD	gaggcgcgccacgcgtcggattatccataataaaaacaaaac
pCLV3 REV	ccaaccatggacgcgtttttagagagaaagtgactgagtg
pRPS5a FWD	gaggcgcgccacgcgtccataatcgtgagtagatatattactcaac
pRPS5a REV	ccaaccatggacgcgtcggctgtggtgagagaaacag
pUBQ10 FWD	ccaaccatggacgcgtctgttaatcagaaaaactcag
pUBQ10 REV	cctcgaggcgcgccacgcgtagtctagctcaacagagct
trbcS-E9 FWD	gcggccgccaccgcggtggagagctttcgttcgtatcatcg
trbcS-E9 REV	agtgtcgtgctccaccatgttgggttgtcaatcaattggcaagtc
tHSP18.2 FWD	gcggccgccaccgcggtggatatgaagatgaagatgaaa
tHSP18.2 REV	agtgtcgtgctccaccatgttggcttatctttaatcatattcc

Table S3. Terminator sequences

Terminator sequence	Sequence (5'-3')
trbcS-E9 (Addgene:71288)	agagctttcgttcgtatcatcggtttcgacaacgttcgtcaagttcaatgcatcagtttcattgcgcacacaccagaatcctactgagtttgagtattatggcattgggaaaactgtttttcttgtaccatttgttgtgcttgtaatttactgtgttttttattcggttttcgctatcgaactgtgaaatggaaatggatggagaagagttaatgaatgatatggtccttttgttcattctcaaattaatattatttgttttttctcttatttgttgtgtgttgaatttgaaattataagagatatgcaaacattttgttttgagtaaaaatgtgtcaaatcgtggcctctaatgaccgaagttaatatgaggagtaaaacacttgtagttgtaccattatgcttattcactaggcaacaaatatattttcagacctagaaaagctgcaaatgttactgaatacaagtatgtcctcttgtgttttagacatttatgaactttcctttatgtaattttccagaatccttgtcagattctaatcattgctttataattatagttatactcatggatttgtagttgagtatgaaaatatttttttaatgcattttatgacttgccaattgattgacaac
tHSP18.2 (At5G59720)	atatgaagatgaagatgaaatatttggtgtgtcaaataaaaagcttgtgtgcttaagtttgtgtttttttcttggcttgttgtgttatgaatttgtggctttttctaatattaaatgaatgtaagatctcattataatgaataaacaaatgtttctataatccattgtgaatgttttgttggatctcttctgcagcatataactactgtatgtgctatggtatggactatggaatatgattaaagataag
